# Impact of treatment with bevacizumab beyond disease progression: a randomized phase II study of docetaxel with or without bevacizumab after platinum-based chemotherapy plus bevacizumab in patients with advanced nonsquamous non–small cell lung cancer (WJOG 5910L)

**DOI:** 10.1186/1471-2407-12-327

**Published:** 2012-08-01

**Authors:** Masayuki Takeda, Isamu Okamoto, Takeharu Yamanaka, Kazuhiko Nakagawa, Yoichi Nakanishi

**Affiliations:** 1 Department of Medical Oncology, Kinki University Faculty of Medicine, 377-2 Ohno-higashi, Osaka-Sayama, Osaka 589-8511, Japan; 2 Department of Medical Oncology, Kishiwada City Hospital, 1001 Gakuhara-cho, Kishiwada, Osaka 596-8501, Japan; 3 Research Center for Innovative Oncology, National Cancer Center Hospital East, 6-5-1 Kashiwanoha, Kashiwa, Chiba, 277-8577, Japan; 4 Research Institute for Diseases of the Chest, Graduate School of Medical Sciences, Kyushu University, 3-1-1 Maidashi, Higashi-ku, Fukuoka, Fukuoka, 812-8582, Japan

**Keywords:** Bevacizumab, Beyond disease progression, Non-small cell lung cancer

## Abstract

**Background:**

Bevacizumab, a humanized antibody to vascular endothelial growth factor (VEGF), shows clinical activity against human cancer, with its addition to standard chemotherapy having been found to improve outcome in patients with advanced nonsquamous non–small cell lung cancer (NSCLC). However, there have been no evidence-based studies to support the continued use of bevacizumab beyond disease progression in such patients treated with the drug in first-line therapy. We have now designed a randomized phase II trial to examine the clinical benefit and safety of continued bevacizumab treatment in patients with advanced nonsquamous NSCLC whose disease has progressed after first-line treatment with bevacizumab plus a platinum-based doublet.

**Methods/Design:**

WJOG 5910L was designed as a multicenter, open-label, randomized, phase II trial by the West Japan Oncology Group of docetaxel (arm A) versus docetaxel plus bevacizumab (arm B) in patients with recurrent or metatstatic nonsquamous NSCLC whose disease has progressed after first-line treatment with bevacizumab plus a platinum-based doublet. Patients in arm A will receive docetaxel at 60 mg/m^2^ and those in arm B will receive docetaxel at 60 mg/m^2^ plus bevacizumab at 15 mg/kg, with each drug administered on day 1 every 21 days until progression or unacceptable toxicity. The primary endpoint of the study is progression-free survival, with secondary endpoints including response rate, overall survival, and safety, for patients treated in either arm.

**Trial registration:**

UMIN (University Hospital Medical Information Network in Japan) 000004715

## Background

Lung cancer is the most common cause of cancer-related death worldwide, with non–small cell lung cancer (NSCLC) accounting for ~75% of all lung cancer cases [1]. Platinum-based chemotherapy regimens are the standard first-line treatment for individuals with advanced NSCLC, but the efficacy of such regimens has reached a plateau [2]. Both experimental and clinical studies have identified many molecules that contribute to the various biological behaviors of malignant tumors including NSCLC.

Vascular endothelial growth factor (VEGF), an endothelial cell–specific mitogen, is the major regulator of angiogenesis in normal and malignant tissue [3,4]. Increased expression of VEGF has been detected in most types of tumor in humans, including NSCLC, and, in many instances, it is associated with increased risk of recurrence, metastasis, or death [5-8]. Preclinical studies have shown that a murine monoclonal antibody specific for VEGF inhibits the growth of human tumor xenografts when administered alone or together with chemotherapy [9-11]. A humanized variant of this antibody, bevacizumab, has shown clinical activity against human cancer, with its addition to standard chemotherapy having been found to improve outcome in the treatment of individuals with metastatic colorectal cancer (mCRC) [12].

Randomized phase III trials have evaluated the addition of bevacizumab to cytotoxic chemotherapy in chemonaïve patients with advanced NSCLC. ECOG 4599 was the first of these trials and established the combination of bevacizumab and cytotoxic chemotherapy as a new standard of care for eligible patients [13]. This trial randomized 878 patients with NSCLC of stage IIIb or IV to chemotherapy alone (carboplatin and paclitaxel) or the same chemotherapy regimen with bevacizumab. Individuals with squamous cell carcinoma, brain metastases, or hemoptysis and those receiving anticoagulation therapy were excluded. Median overall survival (OS) increased from 10.3 to 12.3 months as a result of the addition of bevacizumab (hazard ratio [HR], 0.79; *P* < 0.001), and median progression-free survival (PFS) increased from 4.5 to 6.2 months (HR, 0.66; *P* < 0.001). Toxicities that showed statistically significant but minimal increases in frequency associated with the addition of bevacizumab included hypertension (6.0 versus 0.7%), hemoptysis (1.9 versus 0.2%), and epistaxis (0.7 versus 0.2%) of grade ≥3 as well as neutropenia of grade 4 (25.5 versus 16.8%) and febrile neutropenia (5.2 versus 2.0%). The AVAiL trial randomized patients to receive cisplatin and gemcitabine alone or together with bevacizumab at a dose of either 7.5 or 15 mg/kg [14]. Although OS was originally selected as the primary endpoint, this was changed to PFS during accrual. Like ECOG 4599, the exclusion criteria of AVAiL were broad: a squamous tumor histology, mixed adenosquamous histology if predominantly squamous, hemoptysis greater than one-half teaspoon of bright red blood per event, tumor-invading or abutting major blood vessels, brain metastases or spinal cord compression, uncontrolled hypertension, thrombotic or hemorrhagic disorders in the prior 6 months, and therapeutic anticoagulation within 10 days of the first dose. The median PFS was improved in both the 7.5 mg/kg group (6.7 months; HR, 0.75; *P* = 0.003) and 15 mg/kg group (6.5 months; HR, 0.82; *P* = 0.03) as compared with the chemotherapy-alone arm (6.1 months). OS was not significantly improved by the addition of bevacizumab in this study.

On the basis of these results, bevacizumab plus cytotoxic chemotherapy has become a standard of care for first-line therapy in a subgroup of patients with advanced NSCLC. However, there are no evidence-based studies to support the use of bevacizumab beyond disease progression in NSCLC patients receiving the antibody as first-line therapy. Preclinical studies have shown that VEGF is expressed throughout the life cycle of a tumor [15,16], and that VEGF inhibition results in marked antitumor effects when the inhibitor is administered throughout tumor development. Rapid tumor revascularization has also been shown to occur after removal of anti-VEGF therapy, suggesting that vascular regrowth may be a normal physiological response to the removal of VEGF inhibition [17,18]. Sustained VEGF inhibition has thus been shown to achieve and maintain tumor regression. Insight into the effect of treatment with bevacizumab beyond disease progression has been provided by the nonrandomized, prospective bevacizumab treatment registry known as the BRiTE Study for mCRC [19]. In this prospective study, the impact of treatment with bevacizumab beyond first progression was examined. A total of 1445 patients manifested disease progression and received either further treatment with bevacizumab (*n* = 642, 44%), treatment other than bevacizumab (*n* = 531, 37%), or no treatment (*n* = 253, 18%) after progression. Despite having a patient population more representative of general CRC patients, the group that continued bevacizumab beyond first tumor progression showed a median OS of 31.8 months (*n* = 642), compared with a median OS of 19.9 months for those patients who received treatment other than bevacizumab (*n* = 531), a value similar to that reported in previous randomized studies. On the basis of these findings, the prospective European AIO 0504 trial was undertaken and is currently under way for examination of the effect of the addition of bevacizumab to fluoropyrimidine-based chemotherapy in patients with mCRC who show disease progression while receiving first-line standard chemotherapy plus bevacizumab.

With this background, the current randomized trial (University Hospital Medical Information Network in Japan [UMIN] 000004715) was designed to evaluate whether the addition of bevacizumab to docetaxel alone (the standard second-line treatment for NSCLC) might improve PFS when administered as second-line treatment in NSCLC patients who have progressed after first-line treatment with bevacizumab plus a platinum-based doublet.

## Methods/Design

### Study design

This open-label, randomized, phase II study of the West Japan Oncology Group (WJOG 5910L) was designed to evaluate the addition of bevacizumab to standard therapy with docetaxel. The primary endpoint of the study is PFS, with secondary endpoints including response rate, OS, and safety, for patients treated with either docetaxel alone or docetaxel plus bevacizumab. The study has been approved by the institutional ethics committee of each participating institution.

### Eligibility criteria

Study entry is limited to patients aged ≥20 years with histologically or cytologically confirmed nonsquamous NSCLC that is either recurrent or metastatic, with documented disease progression after first-line treatment with bevacizumab plus platinum-based doublet chemotherapy, and with an Eastern Cooperative Oncology Group (ECOG) performance status of 0 to 2. A patient who has received pre- or postoperative chemotherapy is eligible if the last administration of the prior adjuvant regimen occurred at least 12 months before the onset of platinum-based chemotherapy plus bevacizumab. A patient with a history of treatment with epidermal growth factor receptor (EGFR) tyrosine kinase inhibitors (gefitinib or erlotinib) before platinum-based chemotherapy plus bevacizumab is also eligible if he or she harbors an *EGFR* mutation. Adequate bone marrow, renal, and liver function is required. A lesion not previously irradiated that is measurable according to the Response Evaluation Criteria in Solid Tumors (RECIST) version 1.1 is required for evaluation of response. All patients must sign informed consent forms approved by the relevant institutional review board.

Exclusion criteria include: prior treatment with docetaxel; active or recent history of hemoptysis (at least one-half teaspoon of bright red blood per event); central nervous system metastases; active thrombosis or embolism; serious infection; serious uncontrolled medical conditions including heart disease, diabetes, or hypertension; uncontrolled effusion (pleural, peritoneal, or pericardial effusion requiring drainage for symptom management); major surgery within 4 weeks prior to registration; minor surgical procedures, radiation therapy, or transfusion within the previous 14 days; evidence of interstitial pneumonitis or pulmonary fibrosis on the baseline chest x-ray; pregnancy or lactation; and a history of cancer within the previous 5 years.

### Patient registration

After eligibility criteria are confirmed and informed consent obtained, eligible patients are registered and the planned treatment is initiated by investigators. The accrual began in February 2011 and is to continue for 2 years.

### Treatment plan

**Figure 1:**
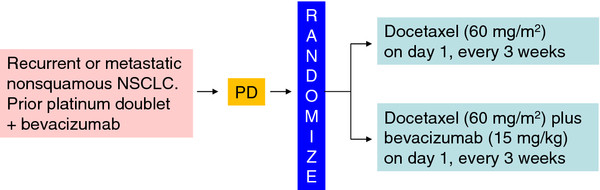
**WJOG 5910L treatment design.** PD, progressive disease.

**Figure 2:**
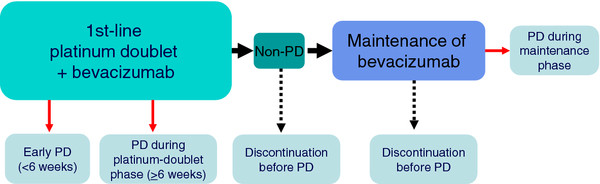
**Disease course patterns for first-line chemotherapy.** Red arrows indicate discontinuation of first-line treatment because of PD; broken black arrows indicate discontinuation before PD.

In this multicenter phase II trial, patients are randomly assigned on a 1:1 basis to docetaxel or docetaxel plus bevacizumab (Figure 1). The study focuses on the outcome of a second bevacizumab-based line of treatment, seeking clinical predictors that might help identify patients likely to benefit from bevacizumab therapy beyond progression (Figure 2). Stratification factors were thus chosen on the basis of the hypotheses that patients who show progressive disease during first-line therapy are unlikely to benefit from second-line therapy and that bevacizumab as a maintenance therapy after treatment with bevacizumab plus a platinum-based doublet until disease progression may augment the efficacy of chemotherapy in second-line treatment. Random assignment was stratified by (i) baseline ECOG performance status (0 or 1 versus 2), (ii) history of treatment with EGFR tyrosine kinase inhibitors (gefitinib or erlotinib versus neither), (iii) duration of treatment with bevacizumab plus a platinum-based doublet (<6 versus ≥6 weeks), and (iv) time to disease progression after the last bevacizumab administration of the first-line treatment (<3 versus ≥3 weeks). Patients treated in arm A will receive docetaxel (60 mg/m^2^) and those in arm B will receive docetaxel (60 mg/m^2^) plus bevacizumab (15 mg/kg) on day 1 every 21 days until progression or unacceptable toxicity. Patients in arm B are allowed to receive docetaxel alone as a result of bevacizumab-related toxicity, but they are not allowed to receive bevacizumab alone. A computed tomography (CT) or magnetic resonance imaging (MRI) scan of the brain, CT scans of the chest and abdomen, bone scan or positron emission tomography (PET) scan, and an electrocardiogram are required before onset of the study treatment. Patients undergo tumor assessment at baseline, every 4 weeks during the first 12 weeks, and every 6 weeks thereafter. Adverse events are recorded based on the National Cancer Institute Common Toxicity Criteria (version 4.0).

### Statistical considerations

The primary and secondary efficacy endpoint analysis will be performed with the intent-to-treat population. The emphasis of the efficacy analysis will be on estimating the size of the difference in treatment effect between arm A and arm B. The sample size was calculated on the basis of hypothesis testing in terms of PFS, which was defined as the time from registration until disease progression or death, whichever occurs earlier. Patients who have not experienced progression or death by the end of follow-up for the study will be censored on the date of the last tumor assessment. The trial is based on a randomized phase II screening design as described previously [20]. A previous randomized phase II study evaluated the efficacy of chemotherapy with or without bevacizumab in patients with advanced nonsquamous NSCLC who were treated with first-line chemotherapy without bevacizumab. The primary endpoint of PFS tended to be longer with chemotherapy plus bevacizumab than for chemotherapy alone (HR, 0.66; 95% confidence interval, 0.38 to 1.16) [21]. Accordingly, the present trial is designed as a one-sided test to detect a ≥30% reduction in the HR associated with PFS favoring the experimental arm with a type I error (alpha) rate of 0.20. The median PFS for docetaxel alone is estimated to be 2.0 months based on the results of previous phase III trials. According to these parameters, a total of 90 or more events (total from both arms combined) is required to achieve a statistical power of >80%. Taking into account patients who prove to be ineligible or who are lost to follow-up, the sample size is planned as 100 patients.

## Conclusion

The WJOG 5910L trial is designed to examine the clinical benefit and safety of continued bevacizumab treatment beyond first disease progression in patients with advanced nonsquamous NSCLC whose disease has progressed after first-line treatment with bevacizumab plus a platinum-based doublet. The information obtained by the study may prompt early completion of the planned patient accrual.

## Competing interests

The authors declare that they have no competing interests.

## Author Contributions

M.T. and I.O. developed the study concept and initiated the project. K.N. and Y.N. coordinated the study concept and protocol design. T.Y. is responsible for statistical analysis. All authors have read and approved the final manuscript.

## Pre-publication history

The pre-publication history for this paper can be accessed here:

http://www.biomedcentral.com/1471-2407/12/327/prepub
